# Expression and Function of Macrophage Migration Inhibitory Factor (MIF) in Melioidosis

**DOI:** 10.1371/journal.pntd.0000605

**Published:** 2010-02-16

**Authors:** W. Joost Wiersinga, Thierry Calandra, Liesbeth M. Kager, Gerritje J. W. van der Windt, Thierry Roger, Didier le Roy, Sandrine Florquin, Sharon J. Peacock, Fred C. G. J. Sweep, Tom van der Poll

**Affiliations:** 1 Center for Infection and Immunity Amsterdam (CINIMA), Academic Medical Center, Amsterdam, The Netherlands; 2 Center for Experimental and Molecular Medicine (CEMM), Academic Medical Center, Amsterdam, The Netherlands; 3 Infectious Diseases Service, Department of Medicine, Centre Hospitalier Universitaire Vaudois and University of Lausanne, Lausanne, Switzerland; 4 Department of Pathology, Academic Medical Center, Amsterdam, The Netherlands; 5 Mahidol-Oxford Tropical Medicine Research Unit, Faculty of Tropical Medicine, Mahidol University, Bangkok, Thailand; 6 Center for Clinical Vaccinology and Tropical Medicine, Nuffield Department of Clinical Medicine, University of Oxford, Oxford, United Kingdom; 7 Chemical Endocrinology, Radboud University Nijmegen Medical Centre, Nijmegen, The Netherlands; Weill Medical College of Cornell University, United States of America

## Abstract

**Background:**

Macrophage migration inhibitory factor (MIF) has emerged as a pivotal mediator of innate immunity and has been shown to be an important effector molecule in severe sepsis. Melioidosis, caused by *Burkholderia pseudomallei*, is an important cause of community-acquired sepsis in Southeast-Asia. We aimed to characterize the expression and function of MIF in melioidosis.

**Methodology and Principal Findings:**

MIF expression was determined in leukocytes and plasma from 34 melioidosis patients and 32 controls, and in mice infected with *B. pseudomallei*. MIF function was investigated in experimental murine melioidosis using anti-MIF antibodies and recombinant MIF. Patients demonstrated markedly increased MIF mRNA leukocyte and MIF plasma concentrations. Elevated MIF concentrations were associated with mortality. Mice inoculated intranasally with *B. pseudomallei* displayed a robust increase in pulmonary and systemic MIF expression. Anti-MIF treated mice showed lower bacterial loads in their lungs upon infection with a low inoculum. Conversely, mice treated with recombinant MIF displayed a modestly impaired clearance of *B. pseudomallei*. MIF exerted no direct effects on bacterial outgrowth or phagocytosis of *B. pseudomallei*.

**Conclusions:**

MIF concentrations are markedly elevated during clinical melioidosis and correlate with patients' outcomes. In experimental melioidosis MIF impaired antibacterial defense.

## Author Summary

Melioidosis is a severe tropical infection caused by the bacterium *Burkholderia pseudomallei*. *B. pseudomallei* is the major cause of community-acquired septicemia in northeast Thailand with a mortality rate in severe cases of around 40% Little is known, however, about the mechanisms of the host defense to *B. pseudomallei* infection. Macrophage migration inhibitory factor (MIF) is a proinflammatory cytokine that has emerged as an important mediator of the host defense in severe bacterial infections. In this article, we studied the expression and function of MIF both in patients with melioidosis and in mice during experimental melioidosis. We found that MIF concentrations were elevated in patients with melioidosis. Furthermore, high MIF concentrations are associated with poor outcome in patients with melioidosis. Also, in mice with experimentally induced melioidosis, we observed an upregulation of MIF concentrations. Furthermore, mice with melioidosis that were treated with a MIF blocking treatment showed lower bacterial counts in their lungs during infection. In conclusion, MIF seems to impair host defense mechanisms during melioidosis.

## Introduction

Macrophage migration inhibitory factor (MIF) was one of the first cytokines to be discovered almost half a century ago [1]–[4]. Since then MIF has emerged as a pivotal mediator of innate immunity in various inflammatory diseases such as rheumatoid arthritis and atherosclerosis [5],[6] and is considered to be an integral component of the host antimicrobial alarm system [4],[7]. MIF, a classical proinflammatory cytokine, is constitutively expressed by many tissues with environmental contact such as the lung and the gastrointestinal tract, and by numerous cell types, among others T- and B-lymphocytes, monocytes and macrophages [4]. MIF-deficient macrophages are hyporesponsive to lipopolysaccharide (LPS) due to a down-regulation of Toll-like receptor (TLR)-4 [8],[9]. In line, MIF knockout mice were resistant to LPS induced toxic shock [8]–[10]. Recently it was shown that blood concentrations of MIF are elevated in patients with sepsis and able to predict early mortality [11]–[14]. Similarly, MIF is increased in patients with meningococcal disease and highest in the presence of shock [15]. Excitingly, treatment with anti-MIF antibodies protected mice from lethal peritonitis induced by *Escherichia coli* or cecal ligation and puncture (CLP) [16]. Furthermore, ISO-1 and OXIM-11, new small molecule inhibitors of MIF, offered significant protection to mice from CLP-induced sepsis [17],[18]. These data identified MIF as a potential mediator of lethality following abdominal sepsis.

In Southeast-Asia and Northern-Australia the gram-negative bacillus *Burkholderia pseudomallei* is an important cause of community-acquired sepsis [19],[20]. More than half of these cases of melioidosis, as this severe infection is named, habitually presents with pneumonia, frequently associated with bacterial dissemination to distant sites [19]–[21]. In the present study we aimed to characterize the expression and function of MIF in melioidosis. For this we analysed MIF expression patterns in patients with melioidosis and in a mouse model of *B. pseudomallei* infection. MIF function was investigated in experimental murine melioidosis using anti-MIF antibodies and recombinant MIF.

## Methods

### Ethics statement

The patient study was approved by both the Ministry of Public Health, Royal Government of Thailand and the Oxford Tropical Research Ethics Committee, University of Oxford, England. We obtained written informed consent from all subjects before the study. The Animal Care and Use of Committee of the University of Amsterdam approved all murine experiments.

### Patients

We included 34 individuals with sepsis caused by *B. pseudomallei* and 32 healthy controls in this study. Individuals were recruited prospectively at Sapprasithiprasong Hospital, Ubon Ratchathani, Thailand in 2004. Sepsis due to melioidosis was defined as culture positivity for *B. pseudomallei* from any clinical sample plus a systemic inflammatory response syndrome (SIRS) [22]. Study design and subjects have been described in detail [23].

### Human plasma MIF and MIF mRNA measurements

Human MIF was measured by ELISA, as described elsewhere [24]. In addition, MIF mRNA levels were measured as follows. Heparin blood samples were drawn from an antecubital vein and immediately put on ice. Leukocytes were isolated using erylysis buffer, dissolved in Trizol and stored at –80°C. Thereafter, RNA was isolated and analyzed by multiplex ligation-dependent probe amplification (MLPA) as described [25],[26] (MRC-Holland, Amsterdam, the Netherlands). Levels of mRNA were expressed as a normalized ratio of the peak area divided by the peak area of the β2 microglobulin (B2M) gene [25].

### Murine melioidosis

Male C57BL/6 mice (age 8–10 weeks) were purchased from Harlan Sprague Dawley Inc. (Horst, The Netherlands). Age-matched animals were used in each experiment. For the inoculum, *B. pseudomallei* strain 1026b, kindly provided by Dr. Don Woods [27],[28], was used and prepared as described [23], [29]–[31]. Pneumonia was induced by intranasal inoculation of a 50 µl (5×10^1^, 2.5×10^2^ or 7.5×10^2^ colony forming units (CFU)/50 µl) bacterial suspension. 48 hours after infection, mice were anesthetized and sacrificed by bleeding from the vena cava inferior [23],[30],[32]. CFUs were determined from serial dilutions of organ homogenates as described [23], [29]–[31]. In some experiments mice were injected intraperitoneally with 2 mg of anti-MIF or non-immune IgG 2 hours before bacterial inoculation or with 50 µg recombinant mouse MIF or control buffer at the onset of infection as described previously [16],[33],[34]. Rabbit polyclonal anti-MIF and recombinant MIF were generated as described [16],[34].

### Murine assays

The ELISA for mMIF developed according to the 4-span approach was used as described in detail [35]. Tumor necrosis factor (TNF)-α, interferon (IFN)-γ, interleukin (IL)-6, IL-10 and IL-12p70 were determined using a cytometric bead array (CBA) multiplex assay in accordance with the manufacturer's instructions (BD Biosciences, San Jose, CA).

### MIF immunochemistry and histologic examination

Four-µm thick lung tissue sections were sampled 48 hours after infection and mounted on aminopropylmethoxysilane-coated glass slides, deparaffinized in xylol, taken through to absolute alcohol and blocked for endogenous peroxidase with 0.1% hydrogen peroxide in methanol. They were boiled in 10 mM citrate buffer in a microwave oven and rinsed in Tris-buffered saline (TBS). To reduce non-specific binding, sections were incubated in normal goat serum (Pel-Freez Biologicals, Rogers, AK) 1∶30 in TBS. After 40-minutes incubation with polyclonal rabbit anti-MIF purified IgG diluted 1∶200 in TBS containing 2% bovine serum albumin (final immunoglobulin concentration: 25 mg/l), the sections were incubated with biotinylated goat anti-rabbit IgG (Vector, Burlingame, CA) diluted 1∶400 and then with ABC-peroxidase complex solution (Vector). Peroxidase activity was revealed with 5-5′-diaminobenzidine as chromogen and the sections were counterstained in Meyer's acid-free hematoxylin. As a negative control, the primary antibody was replaced by pre-immune rabbit purified IgG. Furthermore, to score inflammation, lung and livers from infected mice were harvested 48 hours after infection, fixed in 10%-formalin and embedded in paraffin. Four µm sections were stained with hematoxylin and eosin and analyzed by a pathologist blinded for groups exactly as described previously [23].

### Effect of MIF on bacterial growth *in vitro*

*B. pseudomallei* strain 1026b was used and prepared as described above. In short, *B. pseudomallei* at concentrations from 3×10^3^–3×10^6^ CFU/ml was grown in the presence of recombinant MIF (dose range from 5 to 50 µg/ml) diluted in LB-growth medium.

### Phagocytosis

Phagocytosis was evaluated as described [36],[37]. Heat-killed *B. pseudomallei* was labeled with carboxyfluorescein-diacetate-succinimidyl-ester (CFSE dye, Invitrogen, Breda, The Netherlands). Peritoneal macrophages (derived from 5 different mice per group) were incubated with CFSE-labeled *B. pseudomallei* (2.5×10^7^ CFU/ml) for 0, 60 and 120 minutes. Phagocytosis was stopped by placing cells on ice; thereafter cells were washed in PBS and suspended in Quenching solution (Orpegen, Heidelberg, Germany). To determine the neutrophil phagocytosis capacity, 50 µl of whole blood was incubated with bacteria after which cells were suspended in Quenching solution, incubated in FACS lysis/fix solution (BecktonDickinson) and neutrophils were labeled using anti-Gr-1-PE (Pharmingen). Phagocytosis was determined using FACS.

### Statistical analysis

Values are expressed as means ± standard error of the mean (SEM). Differences between groups were analyzed by Mann-Whitney U test or Kruskal-Wallis analysis with Dunn's posthoc test where appropriate. For survival analysis, Kaplan-Meier analysis followed by log rank test was performed. These analyses were performed using GraphPad Prism version 4.00, GraphPad Software (San Diego, CA). Values of *P*<0.05 were considered statistically significant.

## Results

### MIF expression is elevated in patients with melioidosis and correlates with poor outcome

**Figure 1:**
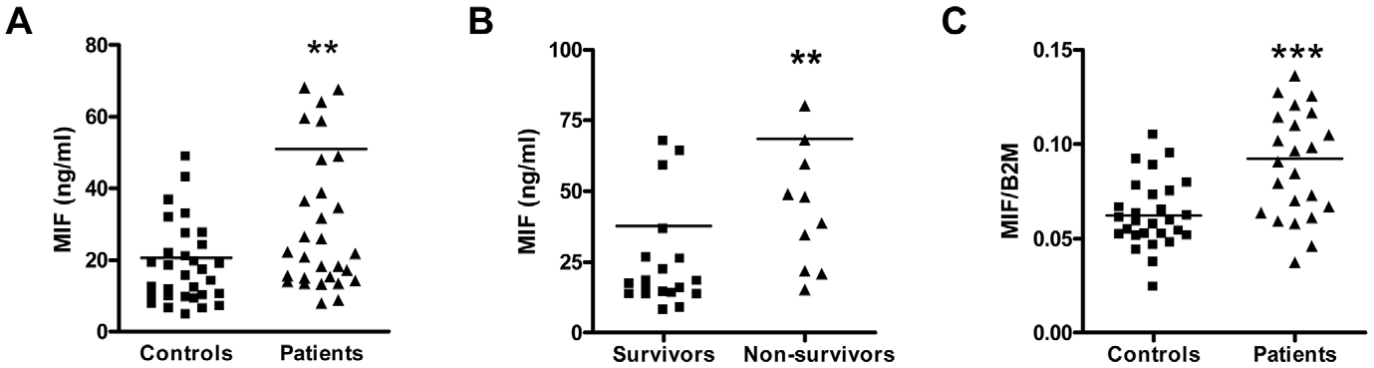
MIF plasma and leukocyte mRNA levels are elevated in melioidosis patients and correlate with poor outcome. In patients (▴, n = 34) with melioidosis, strongly increased plasma concentrations of plasma MIF were present on admission when compared to healthy controls (▪, n = 32) (**A**). Patients who went on to die from melioidosis had higher MIF plasma concentrations on admission than patients who survived (**B**). MIF mRNA was strongly upregulated in the peripheral blood leukocytes of melioidosis patients compared to healthy controls (**C**). B2M: β2 microglobulin. ** p<0.01; *** p<0.001.

To obtain an insight into MIF expression during melioidosis, we first measured MIF in plasma from 34 patients with culture proven *B. pseudomallei* infection and in plasma from 32 local healthy controls. The mortality rate in this cohort of patients was 44%. MIF was markedly elevated in melioidosis patients with mean plasma concentrations that were approximately 2-fold higher than in those of healthy subjects (Figure 1*A, P*<0.01). Plasma concentrations of MIF were associated with an adverse outcome: on admission patients who went on to had higher MIF concentrations than those who survived (Figure 1*B, P*<0.01). In line, MIF mRNA levels were significantly higher in peripheral blood leukocytes from patients than in leukocytes from healthy controls (Figure 1*C, P*<0.001).

### Increased MIF expression in the pulmonary compartment during experimental pneumonia-derived melioidosis

**Figure 2:**
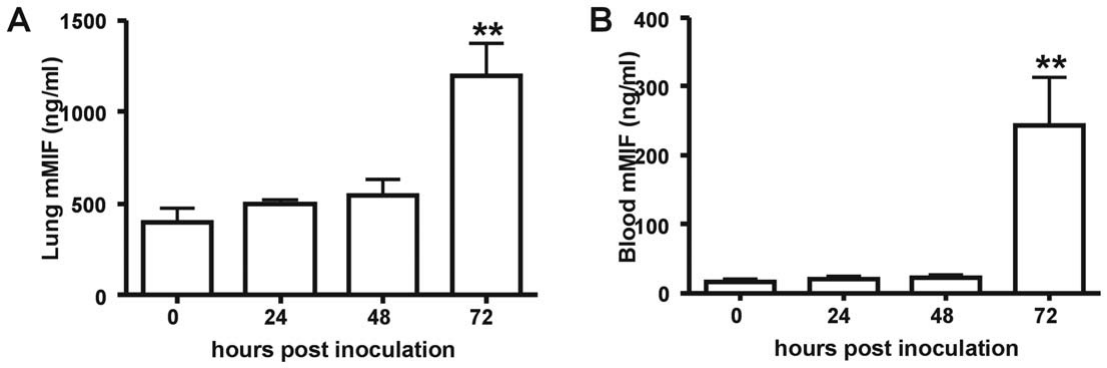
Increased MIF concentrations during experimental melioidosis in mice. Pulmonary MIF expression was strongly increased 72 hours after intranasal inoculation with *B. pseudomallei* (**A**). Plasma MIF concentrations remained constant during the first two days of infection, followed by a steep increase over time (**B**). Data are means ± SEM of 5 mice per group at each time point. ** p<0.01 versus t = 0.

**Figure 3:**
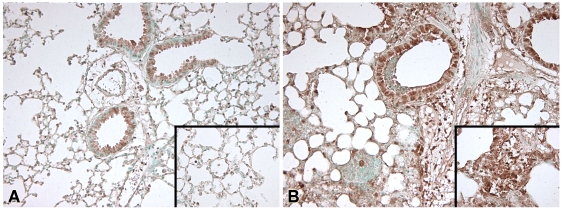
Immunostaining for MIF in the lungs of mice. Positive immunostaining for MIF in lung tissue was observed in non-infected control animals of bronchial epithelial cells and alveolar macrophages (**A**). 48 hours after infection with *B. pseudomallei* there was a marked increase in immunostaining of the epithelial submucosa, bronchial epithelial cells and inflammatory cells, most notably of alveolar macrophages (**B**). Magnification ×10, insets ×20.

Since the majority of severe melioidosis cases presents with pneumonia with bacterial dissemination to distant body sites [19]–[21] and considering the fact that it is not feasible to study MIF expression at tissue level in patients with melioidosis, we used a well-established murine model of pneumonia-derived melioidosis in which mice are intranasally infected with *B. pseudomallei* [23],[29],[31]. In agreement with the data obtained in patients with melioidosis, infected mice showed an abundant upregulation of MIF expression, both in the pulmonary and systemic compartment (Figure 2, both *P*<0.01). Immunohistochemical staining of lung tissue was performed to further identify the distribution of MIF expression during melioidosis. Positive immunostaining for MIF was observed in untreated control animals in alveolar macrophages and within the bronchial epithelium (Figure 3*A*). Granulocytes did not stain positive for MIF. After infection with *B. pseudomallei* there was a marked increase in immunostaining of the epithelial submucosa, bronchial epithelial cells and inflammatory cells, most notably of alveolar macrophages (Figure 3*B*).

### Effect of recombinant MIF treatment on bacterial outgrowth

**Figure 4:**
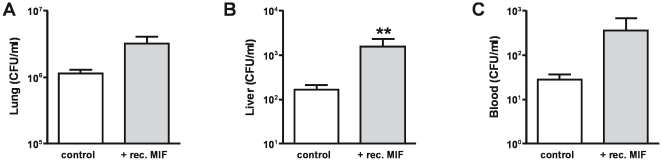
Effect of recombinant MIF on bacterial clearance. Mice, treated with control buffer (white bars) or recombinant MIF (grey bars) and inoculated with 2.5×10^2^ CFU *B. pseudomallei* intranasally, were analysed for bacterial outgrowth in the lungs (**A**), liver (**B**) and blood (**C**) 48 hours later. Data represent mean ± SEM of n = 8 mice per group; ** p<0.01.

To obtain a first insight into the function of MIF during experimental melioidosis, we treated mice infected with 2.5×10^2^ CFU *B. pseudomallei* mice with 50 µg recombinant MIF using a dose similar to that used previously in an experimental septic shock model [16],[33]. Treatment of mice with recombinant MIF at the time of infection resulted in increased MIF concentrations in lung homogenates 48 hours later (from 33±1.3 to 729±56.6 ng/ml; *P*<0.001). Mice were sacrificed 48 hours after inoculation to determine bacterial loads in lungs (the primary site of the infection), liver and blood (to evaluate to which extent the infection disseminated to distant body sites) (Figure 4). Relative to infected but non-treated controls, mice treated with recombinant MIF displayed almost 10-fold higher bacterial loads in the liver (Figure 4, *P*<0.01). In addition, a clear trend was seen towards higher bacterial loads in the pulmonary and systemic compartments of recombinant MIF treated mice, although the differences with control mice did not reach statistical significance (Figure 4).

### Effect of anti-MIF treatment on bacterial outgrowth

**Figure 5:**
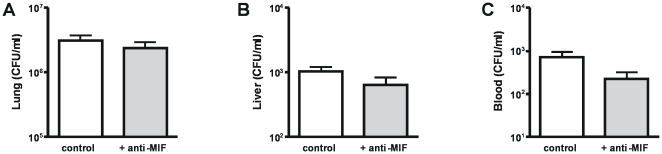
Effect of anti-MIF antibodies on bacterial clearance. Mice were treated with non-immune IgG control (white bars) or anti-MIF antibodies (grey bars) and inoculated with 2.5×10^2^ CFU *B. pseudomallei* intranasally after which bacterial outgrowth in the lungs (**A**), liver (**B**) and blood (**C**) was analysed 48 hours later. Data represent mean ± SEM of n = 8 mice per group.

**Figure 6:**
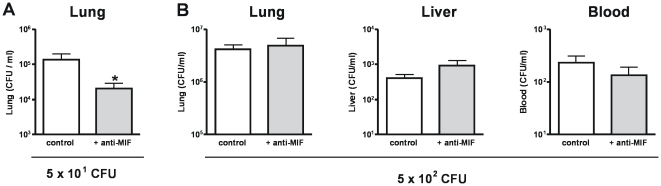
Effect of anti-MIF treatment on bacterial clearance is dependent on the inoculum size. Mice were treated with non-immune IgG control (white bars) or anti-MIF antibodies (grey bars) and inoculated with 5×10^1^ CFU (**A**) or 5×10^2^ CFU (**B**) *B. pseudomallei* intranasally after which bacterial outgrowth in the lungs, liver and blood was analysed 48 hours later. No bacterial outgrowth in the liver and no bacteremia was observed in any of the mice inoculated with 5×10^1^ CFU *B. pseudomallei*. Data represent mean ± SEM of n = 8 mice per group; * p<0.05.

**Figure 7:**
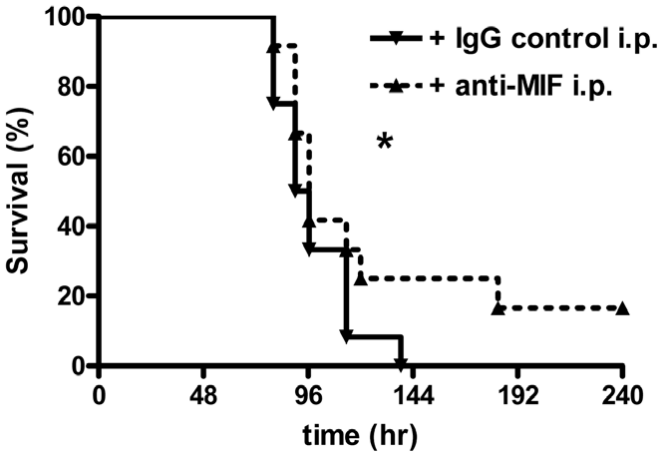
Effect of treatment with anti-MIF antibodies on survival of mice infected with *B. pseudomallei*. Mice were injected intraperitoneally with 2 mg of anti-MIF (straight line with downward arrow) or non-immune control IgG (dashed line with upward arrow) 2 hours before inoculation with 5.0×10^2^ CFU of *B. pseudomallei* intranasally; n = 12 mice per group; * p<0.05.

Having found that administration of supra physiological doses of MIF results in a partially impaired bacterial clearance during experimental melioidosis, we next hypothesized that treatment with anti-MIF antibodies would result in decreased bacterial outgrowth and performed the reverse experiment by examining the effect of anti-MIF treatment. Therefore, before inoculating mice with *B. pseudomallei*, we injected mice with anti-MIF antibodies using a dosing schedule previously found be protective in a mouse model of *E. coli* or CLP-induced peritonitis [16],[33]. To evaluate whether anti-MIF treatment interferes with bacterial clearance, we first determined bacterial loads 48 hours after infection with an inoculum of 2.5×10^2^ CFU *B. pseudomallei* (Figure 5). At this dose no significant differences in bacterial outgrowth in either lungs, liver or blood were observed. To determine whether the effect of anti-MIF therapy is dependent on the size of the infectious dose, we next infected mice with a higher (5×10^2^ CFU *B. pseudomallei*) and lower (5×10^1^ CFU *B. pseudomallei*) inoculum (Figure 6). At the highest dose no effect of anti-MIF treatment was seen on the bacterial outgrowth in the lungs, liver or blood (Figure 6*B*). However, at the lowest inoculum mice treated with anti-MIF had almost 10-fold less *B. pseudomallei* CFU in their lungs compared to control mice (Figure 6*A, P*<0.05). With this low inoculum, none of the mice showed positive *Burkholderia* cultures in liver or blood, suggesting that anti-MIF treatment inhibits the growth of *B. pseudomallei* in the lungs after infection with a relatively low bacterial dose. Lastly, we performed a survival experiment in which mice were injected intraperitoneally with 2 mg of anti-MIF or non-immune control IgG 2 hours before intranasal inoculation with *B. pseudomallei*. In accordance with the modest protective effect of anti-MIF treatment on bacterial outgrowth a limited survival advantage was seen in the anti-MIF treated group (Figure 7).

### Influence of anti-MIF and recombinant MIF on the inflammatory response

Since cytokines are important regulators of the inflammatory response to acute lower respiratory tract infection [38] and given the observation that protective anti-MIF treatment reduced TNFα concentrations in mouse model of sepsis induced by *E. coli* or CLP [16],[33], we measured the concentrations of TNFα, IL-6, IL-10, IL-12 and IFNγ in lung homogenates and plasma obtained 48 after infection with 2.5×10^2^ CFU *B. pseudomallei* (**Table 1**). Anti-MIF treatment did not influence pulmonary cytokine concentrations in our model of experimental melioidosis (**Table 1**). Previously it was shown that plasma TNFα concentrations induced by LPS were lower in MIF-deficient mice compared to wild-type mice [10],[33]. However also in plasma no differences in TNFα or IL-6, IL-10, IL-12 and IFNγ concentrations were seen between anti-MIF treated and control mice after inoculation with *B. pseudomallei* (data not shown). In addition treatment with 50 µg recombinant MIF did not influence cytokine concentrations in either the pulmonary (**Table 1**) or systemic compartment (data not shown). Considering that MIF is regarded as an important proinflammatory mediator, we determined whether modulation of MIF concentrations could have an effect on organ inflammation during experimental melioidosis. Therefore, we performed histopathological analyses of lung and liver tissues in control mice and mice treated with anti-MIF or recombinant MIF and infected with *B. pseudomallei*. Although all mice showed evidence of inflammation as characterized by diffuse infiltrates, interstitial inflammation and bronchitis there were no differences in total organ histopathological scores between groups (data not shown).

**Table 1:** Cytokine concentrations (pg/ml) in lung homogenates of mice treated with anti-MIF antibodies or recombinant MIF during experimental melioidosis.

Cytokine	Non-immune IgG	Anti-MIF mAb	Control	Recombinant MIF
**TNF-α**	2632±524	1217±475	1910±154	2827±652
**IL-6**	2421±255	2461±242	1530±203	2105±459
**IL-10**	97±17	100±8.3	79±13	125±15
**IL-12p70**	91±8.6	108±7.4	116±7.6	116±14
**IFN-γ**	432±198	218±78.4	366±37	311±151

Pulmonary cytokine concentrations after intranasal infection with 2.5×10^2^ CFU *B. pseudomallei*. Mice were sacrificed 48 hours after infection. Data are means ± SEM of eight mice per group per time point. TNF-α: tumor-necrosis-factor (TNF)-α; IL  =  interleukin; IFN-γ  =  Interferon-γ.

### No direct effect of MIF on bacterial outgrowth or phagocytosis

Having found that anti-MIF treated mice showed lower bacterial loads in their lungs upon infection with a low inoculum while mice treated with recombinant MIF displayed a modestly impaired clearance of *B. pseudomallei*, we next wished to determine whether MIF has a direct effect on bacterial outgrowth and/or phagocytosis. Therefore, *B. pseudomallei* (at concentrations from 3×10^3^–3×10^6^ CFU/ml) was grown in the presence of recombinant MIF diluted in the growth medium (dose range from 5 to 50 µg/ml). However, no effects of recombinant MIF on bacterial outgrowth could be observed at any time point (up to 2 hours; data not shown). Lastly, we studied whether MIF contributes to phagocytosis of *B. pseudomallei*. However no effects of anti-MIF treatment on phagocytosis of *B. pseudomallei* by peritoneal macrophages or whole blood neutrophils could be observed (data not shown).

## Discussion

In the present study we aimed to characterize the expression and role for MIF in melioidosis, linking observational studies in patients with culture-proven disease with functional studies in mice in which we modulated the concentrations of MIF during experimentally induced melioidosis. Our study shows that patients with severe melioidosis have strongly increased MIF plasma and MIF mRNA leukocyte levels. High plasma MIF concentrations were associated with mortality. Similarly, mice intranasally inoculated with *B. pseudomallei* displayed a strong increase in pulmonary and systemic MIF expression. The functional role of MIF in our model of experimental melioidosis however was modest given the fact that modulation of MIF levels only moderately influenced the innate immune response towards *B. pseudomallei*. Anti-MIF treatment resulted in a modest survival benefit. Anti-MIF treatment only decreased bacterial outgrowth when mice were inoculated with a low dose of *B. pseudomallei* whereas - conversely - mice treated with recombinant MIF displayed a modestly impaired clearance of *B. pseudomallei.* These data are the first to report on the expression and function of MIF during melioidosis.

MIF expression is increased in a wide variety of infectious diseases, ranging from viral infections, such as Dengue, HIV and West Nile virus infection [39]–[41], malaria [42], tuberculosis [43] and various forms of sepsis [11],[12],[15]. Our study further extends these findings by demonstrating increased plasma and blood leukocyte mRNA levels of MIF in patients with severe melioidosis. Importantly, we demonstrated a strong association between elevated MIF levels and increased mortality. This is in line with a recent study among pediatric and adult patients with severe sepsis or septic shock caused predominantly by *Neisseria meningitides* and other gram-negative bacteria in which elevated MIF levels were shown to be predictive of early mortality [12]. MIF, however, is not always upregulated after acute infection or inflammation. For instance, in children with acute malaria circulating MIF levels were significantly lower compared with healthy, malaria-exposed children [44]. Furthermore, MIF release could not be detected in a human endotoxemia model and is not produced by whole blood cells incubated with LPS [15]. Also in HIV seropositive patients low serum MIF levels were associated with a high 1-month mortality [41]. This further highlights the potential diverse roles MIF can play in the host response against various invading pathogens.

Melioidosis, which is the most common form of community-acquired sepsis in Northern-Australia and Eastern-Thailand, is associated with a mortality of up to 50% in endemic areas [19],[20]. Severe pneumonia with bacterial dissemination to distant body sites is a common presentation of melioidosis [20],[21]. Sepsis caused by *B. pseudomallei* is characterized by a markedly proinflammatory cytokine profile; in the current cohort of patients we have demonstrated increased plasma concentrations of IL-6, IL-8 and IL-18 when compared to controls [31],[45]. In addition, high throughput mRNA profiling in these patients suffering from severe melioidosis furthermore demonstrated increased transcription of a whole array of proinflammatory genes in whole blood leukocytes [26]. In light of the proinflammatory properties attributed to MIF in sepsis, we studied the expression and function of MIF in a well-established mouse model of melioidosis [23],[29],[31]. In line with our patient data and in line with various other murine models of sepsis induced by LPS, *E. coli* or CLP [4],[16],[46], we observed a strong upregulation of MIF expression in both the lungs and blood of mice inoculated with *B. pseudomallei.* However, MIF seems to play a less important role in the innate immune response in melioidosis, which is in contrast with previous studies pointing towards a central role of MIF in other forms of infection. With regard to bacterial infection, the role of MIF has been first studied in abdominal sepsis caused by either intraperitoneal injection of *E. coli* or CLP [9],[16]. In these models, anti-MIF from the same source and administered in the exact same dose protected mice from mortality, reduced TNFα concentrations and diminished bacterial growth. Very recently it was shown that polymorphisms associated with higher MIF expression may have a beneficial effect in community-acquired pneumonia [47]. In addition, modulation of MIF may have therapeutic advantages in treating acute lung injury in patients with acute pancreatitis complicated by bacterial infection [48]. The fact that anti-MIF only has a minor impact on the immune response to *B. pseudomallei* could be related to differences in the primary site of infection and/or differences in the pathogens involved [9],[16]. In this respect it is worthwhile noting that MIF regulates innate immune responses in gram-negative infections through modulation of Toll-like receptor 4 [8]. We obtained recent evidence - counter intuitively for a gram-negative infection - that TLR2 impacts on the immune response of the intact host *in vivo*, whereas TLR4 does not contribute to protective immunity in melioidosis [23]. As such, the minor role of TLR4 in the innate immune response towards *B. pseudomallei* could be an explanation for our present findings revealing an equally limited role for MIF in melioidosis. Interestingly, during murine *Listeria monocytogenes* infection, the elimination of bacteria from the spleen and liver was not affected by anti-MIF antibody although this treatment was able to rescue mice from lethal infection [49].

In reverse experiments we found that treatment of *B. pseudomallei* infected mice with recombinant MIF caused impairment of the bacterial clearance capability. Earlier studies showed that recombinant MIF increased mortality during *E. coli* sepsis when co-injected with bacteria in mice [16],[33],[50]. In these investigations the effect of recombinant MIF on bacterial loads was not reported. These findings imply that increased concentrations of MIF can be harmful in the acute host response against invading bacteria. In this respect it is of interest that during the immune suppressed state which occurs in the late phase of the septic response and which is characterized by a reduced capacity of immune cells to produce proinflammatory cytokines such as TNFα, it was shown that treatment with recombinant MIF could protect animals from bacterial superinfection in a mouse model of CLP-induced peritonitis [51]. This further highlights the potential diverse nature of MIF function during the course of sepsis.

Our study has several limitations. Our observations were done in patients with sepsis caused by *B. pseudomallei* and caution is required when extending these findings to less severe or chronic melioidosis, since we focused on the early acute phase of melioidosis. Furthermore, although our *in vivo* model of melioidosis has been important in elucidating the role of other inflammatory mediators in melioidosis [23],[29],[31], data obtained from a mouse model by definition should be extrapolated to patients with melioidosis with great caution. In addition, it would be of interest to confirm our results in MIF knockout mice, although we consider it less likely that the use of these mice will yield strongly different data in light of the modest differences observed in the different treatment groups. Lastly, obtaining new biological insights from studies using antibodies and recombinant proteins of interest remains a challenge, limited by the notion of considerable cooperation between inflammatory factors involved and extensive redundancy in the host response against invading pathogens [52].

In conclusion, MIF concentrations are markedly increased during melioidosis, and elevated levels correlate with mortality. Although mice with experimentally induced melioidosis showed strongly upregulated expression of MIF in lungs and blood, inhibition of MIF with a specific antibody only modestly influenced the host response. Similarly, administration of recombinant MIF did not strongly impact on the immune response to *B. pseudomallei* infection. These data argue against an important role for MIF in the pathogenesis of melioidosis.
